# A Dictionary Learning Approach for Signal Sampling in Task-Based fMRI for Reduction of Big Data

**DOI:** 10.3389/fninf.2018.00017

**Published:** 2018-04-12

**Authors:** Bao Ge, Xiang Li, Xi Jiang, Yifei Sun, Tianming Liu

**Affiliations:** ^1^Key Laboratory of Modern Teaching Technology, Ministry of Education, Xi'an, China; ^2^School of Physics and Information Technology, Shaanxi Normal University, Xi'an, China; ^3^Cortical Architecture Imaging and Discovery Lab, Department of Computer Science and Bioimaging Research Center, University of Georgia, Athens, GA, United States; ^4^The Clinical Hospital of Chengdu Brain Science Institute, MOE Key Lab for Neuroinformation, School of Life Science and Technology, University of Electronic Science and Technology of China, Chengdu, China

**Keywords:** task-fMRI, sampling, sparse representation, dictionary learning, brain networks

## Abstract

The exponential growth of fMRI big data offers researchers an unprecedented opportunity to explore functional brain networks. However, this opportunity has not been fully explored yet due to the lack of effective and efficient tools for handling such fMRI big data. One major challenge is that computing capabilities still lag behind the growth of large-scale fMRI databases, e.g., it takes many days to perform dictionary learning and sparse coding of whole-brain fMRI data for an fMRI database of average size. Therefore, how to reduce the data size but without losing important information becomes a more and more pressing issue. To address this problem, we propose a signal sampling approach for significant fMRI data reduction before performing structurally-guided dictionary learning and sparse coding of whole brain's fMRI data. We compared the proposed structurally guided sampling method with no sampling, random sampling and uniform sampling schemes, and experiments on the Human Connectome Project (HCP) task fMRI data demonstrated that the proposed method can achieve more than 15 times speed-up without sacrificing the accuracy in identifying task-evoked functional brain networks.

## Introduction

The increasing spatial and temporal resolutions as well as larger sample sizes lead to a rapid increase in the amount of fMRI data. For instance, the ongoing Human Connectome Project (HCP) (Barch et al., 2013) released its task fMRI data with around 240,000 signal time series of a few hundred time points for each of 1,200 subjects. However, the opportunities offered by the availability of fMRI big data from a large number of subjects are accompanied with challenges (Akil et al., 2011; Boubela et al., 2015). In particular, the challenge to address the computational demands associated with the increase in data size becomes clear (Cunningham and Byron, 2014), especially for fMRI big data (Li et al., 2016). Thus, this fMRI big data challenge more and more stresses the significance of data reduction and meaningful information extraction for brain mapping.

To deal with this big data challenge, some existing approaches achieved good performance (Mwangi et al., 2014; Smith et al., 2014), Two popular reduction techniques in neuroimaging are principal component analysis (PCA) (Smith et al., 2014), independent component analysis (ICA) (Calhoun et al., 2008) and its variants. PCA constructs relevant features by linearly transforming correlated variables (e.g., raw voxels in a brain scan) into a smaller number of uncorrelated variables, also known as principal components. ICA is a multivariate data-driven technique which belongs to the broader category of blind-source separation methods used to separate data into underlying independent information components, while ICA separates a set of “mixed signals” (e.g., raw data from an fMRI scan) into a set of independent features. However, these methods have difficulty in reconstructing concurrent interacting functional networks (Friston et al., 1994; Calhoun et al., 2001; Smith et al., 2014), because their implicit assumption is that different components are independent (Smith et al., 2014) or uncorrelated (Calhoun et al., 2001). Recently, dictionary learning and sparse representation (Mairal et al., 2010; Wright et al., 2010) have been explored to represent whole-brain fMRI signals and to reconstruct concurrent functional brain networks (Li et al., 2009, 2012; Lee et al., 2011, 2013; Oikonomou et al., 2012; Lv et al., 2014a; Abolghasemi et al., 2015). Different from ICA and PCA methods, dictionary learning and sparse representation make a sparsity assumption instead of independence or uncorrelation, which is more aligned with the sparseness of neuronal activity property, and promising results have been reported in the literature (Lv et al., 2014a,b). However, these dictionary learning methods still cost significant amount of time and memory space to learn the dictionaries for one brain's single fMRI scan since the input data is huge, for example,a 4-D fMRI data in HCP dataset (Barch et al., 2013) used in this work has about 200 Megabytes with a number of over 10^6^ voxels, each of which contains a time series of hundreds of time points. The computing time cost thus would significantly hamper the wider application of sparse representation methods to larger scale fMRI datasets such as the HCP task fMRI data. Therefore, this challenge urges and motivates us to investigate an efficient signal sampling method in this paper to extract the most representative signals from task fMRI data without losing crucial information for functional network reconstruction but can significantly speed up the computing. Our rationale is that the sampled fMRI signals can statistically, computationally and biologically well represent the original whole-brain fMRI data for concurrent brain network reconstruction based on prior successful applications of sampling methods in the statistical science fields (Rao, 2000; Mahoney, 2011; Meng et al., 2014).

In this paper, we present a structurally guided fMRI signal sampling method for dictionary learning and sparse representation of task fMRI data. Specifically, it effectively extracts the fMRI signals according to the key structural brain information contained in the Dense Individualized and Common Connectivity-based Cortical Landmarks (DICCCOL) system (Zhu et al., 2013). We compared this DICCCOL-based sampling method with no sampling, random sampling, and uniform sampling schemes, and experimental results on the HCP task fMRI data showed that the DICCCOL-based sampling scheme can speed-up the computation by more than 15 times without losing much information. Meanwhile, we learned the common network dictionaries in a group-wise fashion by aggregating the sampled task fMRI signals of multiple subjects into a big data matrix, which further significantly reduced the computation time. This group-wise sampling and aggregation method also contributes to the identification of group-wise consistent functional brain networks across individual subjects. It should be noted that we have proposed a signal sampling and associated dictionary learning strategy for the sparse representation of resting-state fMRI data in our previous studies (Ge et al., 2015) and the major differences between this work and our previous one (Ge et al., 2015) lie in three aspects. First, in this paper, we focus on using signal sampling and sparse coding on task fMRI data, while the data type of the previous work (Ge et al., 2015) was resting state fMRI. Second, the previous dictionary learning step in Ge et al. (2015) learned one dictionary set for each subject, while the current work in this paper learns a common group-wise consistent dictionary set for a population of subjects. Thus, the data input in this work is the aggregated task fMRI signals from the group. Third, with the availability of the abovementioned common dictionary within a group of subjects, we can identify and examine common functional networks easily in each individual subject. The intrinsically established correspondences of these common networks make it possible to examine brain networks at both of individual level and population level (Lv et al., 2015), while the previous work identified the corresponding networks by matching technique without intrinsic correspondence.

## Experiment procedures

### Overview

**Figure 1:**
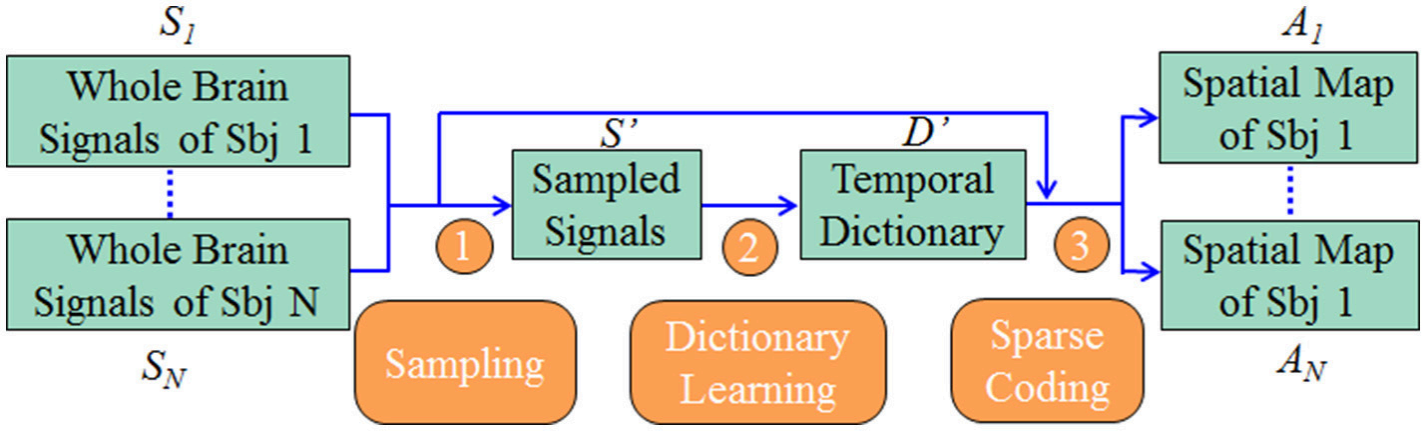
The overview of our computational framework. The sampling step (step 1) includes four types of sampling methods (DICCCOL-based sampling, random sampling, uniform sampling, and no sampling). More details are referred to Dictionary Learning and Sparse Coding to Group-wise DICCCOL-based Dictionary Learning and Sparse Coding.

Our computational framework is shown in Figure 1. First, we sampled the whole brain task fMRI signals using DICCCOL-based sampling, no sampling, random sampling, and uniform sampling, respectively. The sampled signals of all subjects were then aggregated into a big data matrix, after which we applied the online dictionary learning and sparse coding method (Mairal et al., 2010) to learn a group-wise common dictionary shared by all subjects. Finally, we used the common dictionary to sparsely represent each fMRI signal and to identify functional brain networks in each subject.

### Materials and pre-processing

#### Materials

The motor task fMRI and DTI data from the HCP dataset (Barch et al., 2013) were used in our work. There were 6 task designs altogether in the paradigm, in which participants were presented with visual cues instructing them to tap their left or right fingers, squeeze their left or right toes, or move their tongue. The total run time is 284 TRs. Each block of a movement type lasts 12 s (10 movements), and is preceded by a 3 s cue. There are 13 blocks, with 2 of tongue movements, 4 of hand movements (2 right and 2 left), 4 of foot movements (2 right and 2 left) and three 15 s fixation blocks per run. Whole-brain EPI acquisitions were acquired with a 32 channel head coil on a modified 3 T Siemens Skyra with *TR* = 720 ms, *TE* = 33.1 ms, flip angle = 52°, *BW* = 2,290 Hz/Px, in-plane FOV = 208 × 180 mm, 72 slices, 2.0 mm isotropic voxels, with a multi-band acceleration factor of 8, the total run time is 284 TRs. The additional details about DTI and motor task are referred to Barch et al. (2013).

#### Pre-processing

The preprocessing of task fMRI data included skull removal, motion correction, spatial smoothing, temporal pre-whitening, slice time correction, and global drift removal. Preprocessing of the DTI data included skull removal, motion correction, and eddy current correction.

### Dictionary learning and sparse coding

Different from other matrix decomposition methods such as independent component analysis (Smith et al., 2012), principal component analysis (Andersen et al., 1999) and etc., dictionary learning algorithms (Mairal et al., 2010; Lee et al., 2011; Eavani et al., 2012; Yang et al., 2014; Abolghasemi et al., 2015) aim to learn an over-completed dictionary and do not impose that the basis vectors be orthogonal, which better agrees with the current neuroscience perspective, that is, a variety of cortical regions and networks exhibit strong functional diversity and heterogeneity (Kanwisher, 2010; Anderson et al., 2013; Fedorenko et al., 2013). For instance, it was suggested that “areas of the brain that have been associated with language processing appear to be recruited across other cognitive domains” (Gazzaniga, 2004). In addition to the above principle that cortical regions/networks have strong functional diversity and heterogeneity, another important principle is that the number of functional networks that a cortical region is involved in at a specific moment is sparse (Anderson et al., 2013), typically from several to one or two dozen. Therefore, dictionary learning and sparse coding method have been shown to be a powerful tool in constructing functional networks from various types of fMRI signals (Lee et al., 2011; Lv et al., 2014b).

**Figure 2:**
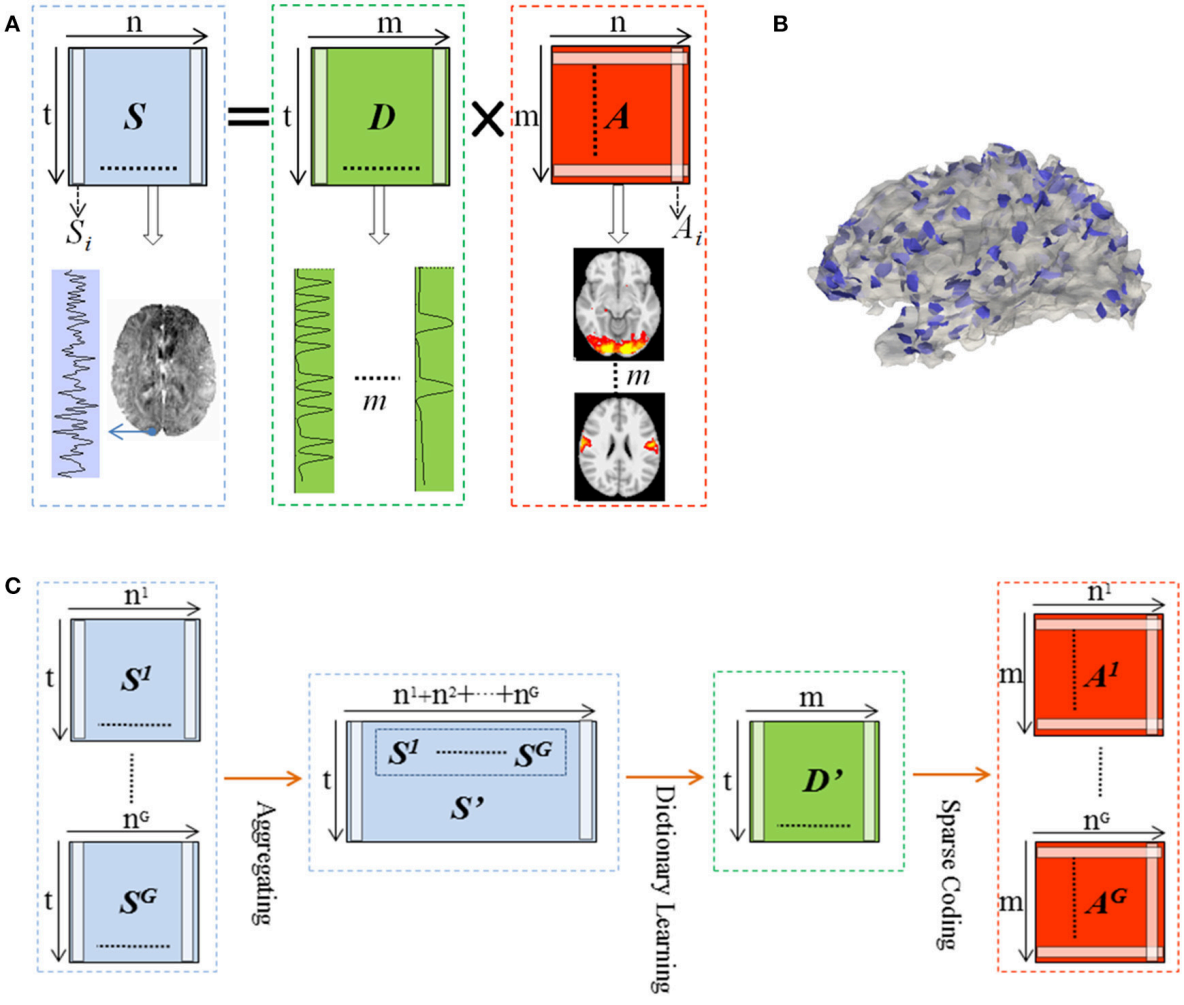
Illustration for the online dictionary learning and sparse representation framework. **(B)** The 2-ring neighborhood DICCCOL sampling. The blue patches show the sampling regions in the whole brain. **(C)** Illustration for group-wise DICCCOL-based dictionary learning and sparse Coding.

We represented whole brain fMRI signals sparsely and reconstructed the task-evoked functional brain networks by the online dictionary learning and sparse coding method (Mairal et al., 2010; Lv et al., 2014b) with the basic premise: the observed fMRI signals are the result of linear combination from the signals of many latent sources, noises and artifacts signals (Biswal and Ulmer, 1999; Calhoun et al., 2008). Generally, the dictionary learning and sparse coding method decomposes an fMRI signal into a group of latent sources (i.e., dictionary) and corresponding combination coefficients, as shown in Figure 2A. That is, given the whole-brain task fMRI signal matrix *S*ϵℝ^*t*×*n*^, we can represent *S* as *S* = *D* × *A*, where *D*ϵℝ^*t*×*m*^ is the dictionary and *A* ϵℝ^*m*×*n*^ is the coefficient matrix. Here, each column of *S* represents a task fMRI signal time series with a time length of *t*, and altogether *n* signals in whole brain; each column in *D* is a learned dictionary atom and m denotes the number of dictionary atoms (i.e., latent sources). For each task fMRI time series *S*_*i*_, it can be represented as linear combination of atoms of dictionary, that is, *S*_*i*_ = *D* × *A*_*i*_, where *A*_*i*_ is i-th column of *A* that gives the sparse weights for the combination, as shown in Figure 2A.

The matrix decomposition is regularized by the *L-*1 norm (i.e., sum of the absolute value of all elements) of matrix *A*, thus adding the sparsity constraint on the number of basis dictionary atoms to represent the input fMRI data. Dictionary matrix *D*_*i,j*_ and its corresponding loading coefficient *A*_*i,j*_ are obtained simultaneously by an iterative optimization process implemented by the online dictionary learning method (Wright et al., 2010), with the loss function defined below:

$$\underset{\text{D}\in {\mathbb{R}}^{\text{t}\times \text{m}},{\text{A}}_{\text{i}}\in {\mathbb{R}}^{\text{m}}}{\min}\frac{1}{2}||S_{i}-DA_{i}|{|}_{F}+\lambda ||A_{i}|{|}_{1}$$

Finally, each row of the *A* matrix characterizes how each dictionary atom contributes to the formation of each functional signal across all voxels, which is the spatial volumetric distributions in the brain that have references to certain dictionary atoms. More details of dictionary learning and sparse coding procedures are referred to Mairal et al. (2010) and Wright et al. (2010).

When *n* (the number of whole brain's signals) is very large, the estimation of *A* and *D* through penalized least squares is very time consuming, especially when we computed the group-wised consistent dictionary which need to load a group of brain's signals in the later step. Therefore, we intended to sample a small proportion of the observations, which were expected to be able to represent whole brain's signals for this matrix decomposition.

### Group-wise DICCCOL-based dictionary learning and sparse coding

It has been demonstrated that DICCCOL provided 358 consistent structural corresponding landmarks across different individual subjects, and they can also be predicted on a new brain (Zhu et al., 2013). The process of generating these landmarks is briefly described as follows. First, a simple strategy is adopted to generate a regular grid initialization of 2056 cortical landmarks for each subject, establishing rough correspondences across subjects by linear registration. The initialization aimed to place a dense map of candidate ROIs distributed over brain cortex. Then the optimization of ROI locations was formulated as an energy minimization problem, which minimized the group-wise non-consistency of DTI-derived fiber shape patterns penetrating candidate ROIs by the following equation,

$$\begin{array}{l} E\;(S_{1},S_{2},\ldots ,S_{m})=\sum E\;(S_{k},S_{l})=\frac{\sum \sum_{i=1}^{n}{\left(T_{ki}-T_{li}\right)}^{2}}{n},\\ \;k\neq l\text{ and }k,l=1,2,\ldots ,m. \end{array}$$

For any two subjects *S*_*k*_ and *S*_*l*_, they are transformed to the corresponding vector format, *T*_*k*_ and *T*_*l*_, of trace-maps. *T*_*ki*_ and *T*_*li*_ are the ith attributes of *T*_*k*_ and *T*_*l*_ respectively. The trace map is a model that describes the shape pattern of the extracted fiber bundle, which reflects the accumulation of the strength of the fiber bundle in different directions and is represented by T. By searching the whole-space of candidate locations, an optimal combination of new landmarks can be found, and exhibited the least group variance of the fiber bundle shape pattern in the training samples. These DICCCOL landmarks were then used as model landmarks to predict new landmarks for a new subject, and the similar optimization step was employed to localize the landmarks so that the dissimilarity of the fiber bundle shape patterns between the candidate and each model landmarks was minimized.

Therefore these DICCCOL landmarks can be considered as the key structural locations of the brain, which were used to sample the whole brain's signals in our work. Specifically, we extended the DICCCOL landmarks to its 2-ring neighborhood on cortical surface (empirically determined) as illustrated in Figure 2B, and then extracted the task fMRI signal of each vertex within the 2-ring regions as the sampled data.

Moreover, we designed the group-wise dictionary learning and sparse coding scheme as follows, shown in Figure 2C. First, we sampled the whole brain's fMRI signals using the specific sampling schemes, such as the DICCCOL-based sampling. Second, we aggregated the sampled signals from a group of subjects into a big data matrix *S*′, and then adopted the online dictionary learning (section Dictionary Learning and Sparse Coding) to learn a group-wise common dictionary *D'* shared by all subjects. That is, *S*′ = *D*′ × *A*′. Now instead of identifying the dictionary using a single subject's single task fMRI data, we store all multiple subjects' data together. Consider a model *S*′ = *D*′*A*′ + *E* where *D*′ is *t* by *m* matrix, *A*′ is a *m* by *n* coefficients matrix. Then we may fit the model using the penalized least squares with *L-*1 penalty,

$$\hat{A^{\prime }}=arg\underset{\text{A}}{\min}{\left|\left|S^{\prime }-D^{\prime }A^{\prime }\right|\right|}^{2}+\lambda |A^{\prime }|.$$

It is well-known that $\hat{A^{\prime }}$ can be efficiently calculated using LARS, as shown in Efron et al. (2004).

Finally, to obtain the sparse representation of whole brain fMRI signals for each subject, we employed the sparse coding step on each subject's whole brain signals matrix *S*_*g*_ based on *D*′, that is, *S*_*g*_ = *D*′ × *A*_*g*_ (as shown in Figure 2C), g is the sequence number of subject. It is noted that since learning *D* and *A* are two separated processes in the online dictionary learning and sparse coding algorithm, we need not to learn *A'* in the second step, which are useless in our work. Therefore, we performed one-time dictionary learning (obtaining *D'*) and one-time sparse coding (obtaining *A*_*g*_), which did not add any more steps and computation burden in the algorithmic pipeline. Here, from a brain science perspective, each dictionary atom represents a functional brain network (Cunningham and Byron, 2014). Afterwards, each row of learned *A*_*g*_ represented by each atom was mapped back to the brain volume for network spatial pattern visualization and characterization. These functional networks are then identified and compared with the task-evoked networks identified from the widely adopted general linear model (GLM) (Friston et al., 1994) method as references.

For no sampling, random sampling and uniform sampling schemes, we performed the above similar procedure to learn the task-evoked networks. The only difference was the aggregated input matrix, which was derived from different sampling schemes. For a fair comparison, we sampled the same number of sampling points in DICCCOL-based sampling for random and uniform samplings, which were respectively corresponding to the numbers of signals of 0-ring, 2-ring, 4-ring and 6-ring DICCCOL sampling, and selected the same set of parameters for all of these three sampling methods.

### Measurements for evaluation and visualization

Since we adopted the learned dictionary and corresponding coefficients to represent the whole brain's fMRI signals, there should be reconstruction/representation errors, which can be computed by the following expression (Mairal et al., 2010):

$$R=\frac{1}{n}\sum_{i=1}^{n}\frac{1}{2}||S_{i}-DA_{i}|{|}_{2}^{2}$$

In order to evaluate the four sampling methods, we compared the activation maps (task-evoked networks) of the sampling methods with that obtained by the GLM method via the Spatial Matching Ratio (SMR), defined as follows:

$$SMR\left(X,T\right)=\frac{\left|X\cap T\right|}{\left|T\right|}$$

where *X* is the spatial activation map from our method, and *T* is the spatial activation map detected by GLM method. |*X* ∩ *T*| and |*T*| are the numbers of voxels in both *X* and *T* and in *T*, respectively.

Different from the previous traditional threshold settings that are constant for visualizing all task-evoked brain networks, here, we designed an adaptive threshold method to visualize each task-evoked brain network. Generally, there are a high threshold and a low threshold to be set for visualizing the activated voxels. We computed the mean value and standard deviation of the activated coefficients matrix, and then used the mean value as the low threshold and sum of them as the high threshold.

## Results

By applying the above four sampling methods on the randomly selected 20 HCP subjects separately, we aggregated the 20 sampled data into one data matrix. Then we performed the dictionary learning and sparse coding according to the procedure in Figure 1. Finally a common dictionary and separated spatial maps were obtained, which will be compared with the task stimulus and GLM derived spatial maps in the following sections. Also, the time cost of four sampling methods will also be evaluated and compared.

### Comparison of temporal dictionary atoms with task stimulus curves

To evaluate and validate the effectiveness of the derived common group-wise dictionaries, we compared them with the task stimulus curves. First, we convolved each of the six task stimulus curve with hemodynamic response function (HRF) using the FSL toolbox in order to compensate the difference between the original input stimulus and output hemodynamic response. Then we selected the six most matched dictionary atoms with each task stimulus by computing the Pearson's Correlation Coefficient. Those identified dictionary atoms can be considered as the bases for representing those fMRI signals which follow the six task stimulus curves.

**Figure 3:**
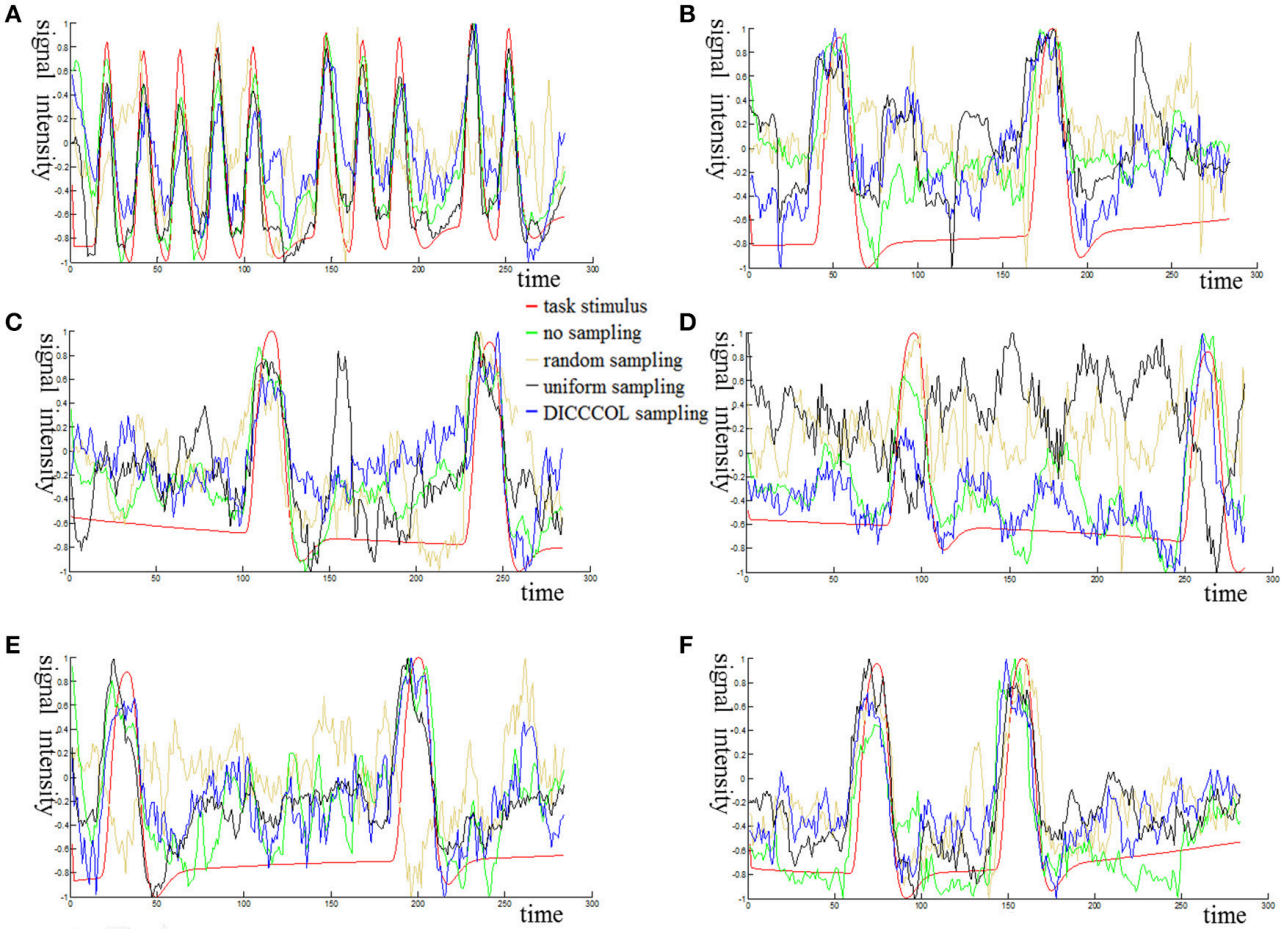
The 6 task stimuli curves and corresponding dictionary atoms **(A–F)** by the four sampling methods. In each sub-figure of **(A–F)**, the red curve denotes a specific task stimulus which serves as a benchmark for comparison. The green, blue, black, and tan curves denote the most matched dictionary atoms derived from no sampling, DICCCOL-based sampling, uniform sampling, and random sampling, respectively.

Figure 3 shows an example of these identified most matched bases using the four sampling methods. It is evident that the blue curve (from the 2-ring neighborhood DICCCOL sampling) and the green one (from no sampling) are more consistent with the red curve (task stimulus), while the black curve (from the uniform sampling) performs slightly worse, especially when we examined Figures 3B–D, that is, the tan curve (the random sampling) is more like a random curve that could not find any relation with the task stimulus by visual inspection. Quantitatively, we calculated their Pearson's correlation coefficients (PCC) with the task stimulus curve (the second row in Table 1A). We can see that the 2-ring DICCCOL sampling and corresponding uniform sampling have better performances than random sampling (the PCCs of 0.74, 0.73, vs. 0.58). Moreover, if we sampled more points, the DICCCOL sampling and uniform sampling resulted in higher PCC. They have almost the same high PCC as no sampling when the number of sampling points reached 14,600 and 26,871. Notably, the 6-ring DICCCOL sampling with 26,871 sampling points even has a higher PCC than no sampling (0.82 vs. 0.81). In order to check when the DICCCOL sampling or other sampling methods have the same performance with no sampling, we made a t-test on PCC and representation error values between no sampling and each other sampling method. As shown in Table 1B, we can see that when we performed the 4-ring DICCCOL sampling with 14,600 sampling points, the 4-ring DICCCOL sampling has no statistical significant difference on PCC value with no sampling. However, all levels of the uniform or random sampling methods have the statistical differences with no sampling. Here, due to limited space, we just show the p-values between the DICCCOL/uniform sampling and no sampling. For representation error, we found that the 2-ring or higher level of DICCCOL sampling had no statistical difference with no sampling, and the uniform sampling can achieve this by U4 or higher level of sampling. So, in general, these results indicated that DICCCOL-based sampling is more effective than other sampling methods and the 4-ring DICCCOL-based sampling is a statistical reasonable choice with the least sampling points.

Table 1**(A)** Quantitative comparison of the four different sampling methods.

**Table:** 

	**W**	**R0**	**U0**	**D0**	**R2**	**U2**	**D2**	**R4**	**U4**	**D4**	**R6**	**U6**	**D6**
**(A)**													
PCC	0.81	0.49	0.71	0.72	0.58	0.73	0.74	0.61	0.79	0.78	0.66	0.75	**0.82**
SMR	0.46	0.11	0.38	0.41	0.22	0.39	0.46	0.31	0.45	0.49	0.31	0.50	**0.52**
Time	1,953	3.7	4.7	4.6	22.5	28.2	54.3	69.7	77.4	76.1	131.2	140.9	138.1
Error	1,8.9	27.6	19.0	19.1	19.3	18.9	19.0	19.2	18.9	19.0	19.1	18.9	18.9
Num	2.4 × 10^5^	358			4,825			14,600			26,871

**Table:** 

	**W vs. D0**	**W vs. D2**	**W vs. D4**	**W vs. D6**	**W vs. U0**	**W vs. U2**	**W vs. U4**	**W vs. U6**
**(B)**								
PCC	0.62	2.97	7.36	90.46	0.03	0.57	3.94	4.10
Error	0.02	5.48	19.89	23.93	0.00	2.49	48.37	10.03

*The second row to the fifth row show the Pearson‘s correlation coefficient between the dictionary atoms of each sampling method and the task stimulus curve, the spatial matching ratio of task-evoked network with that of GLM, the computing time cost, and the representation error of each sampling method, respectively. The sixth row provides the number of sampling points in each circumstance. “W” denotes the whole-brain signals with no sampling, “Dn” represents n-ring DICCCOL-based sampling, “Rn” and “Un” represent random and uniform sampling, respectively, using the same number of points as the n-ring DICCCOL-based sampling. **(B)** The p-values (× 10^−2^) of t-test on PCC and Error. T-test comparisons between no sampling and the DICCCOL/uniform sampling are shown here. The color values denote the p-values < 0.05. Bold values indicate they are the highest values per row*.

### Comparison of spatial task-evoked networks with GLM derived activations

**Figure 4:**
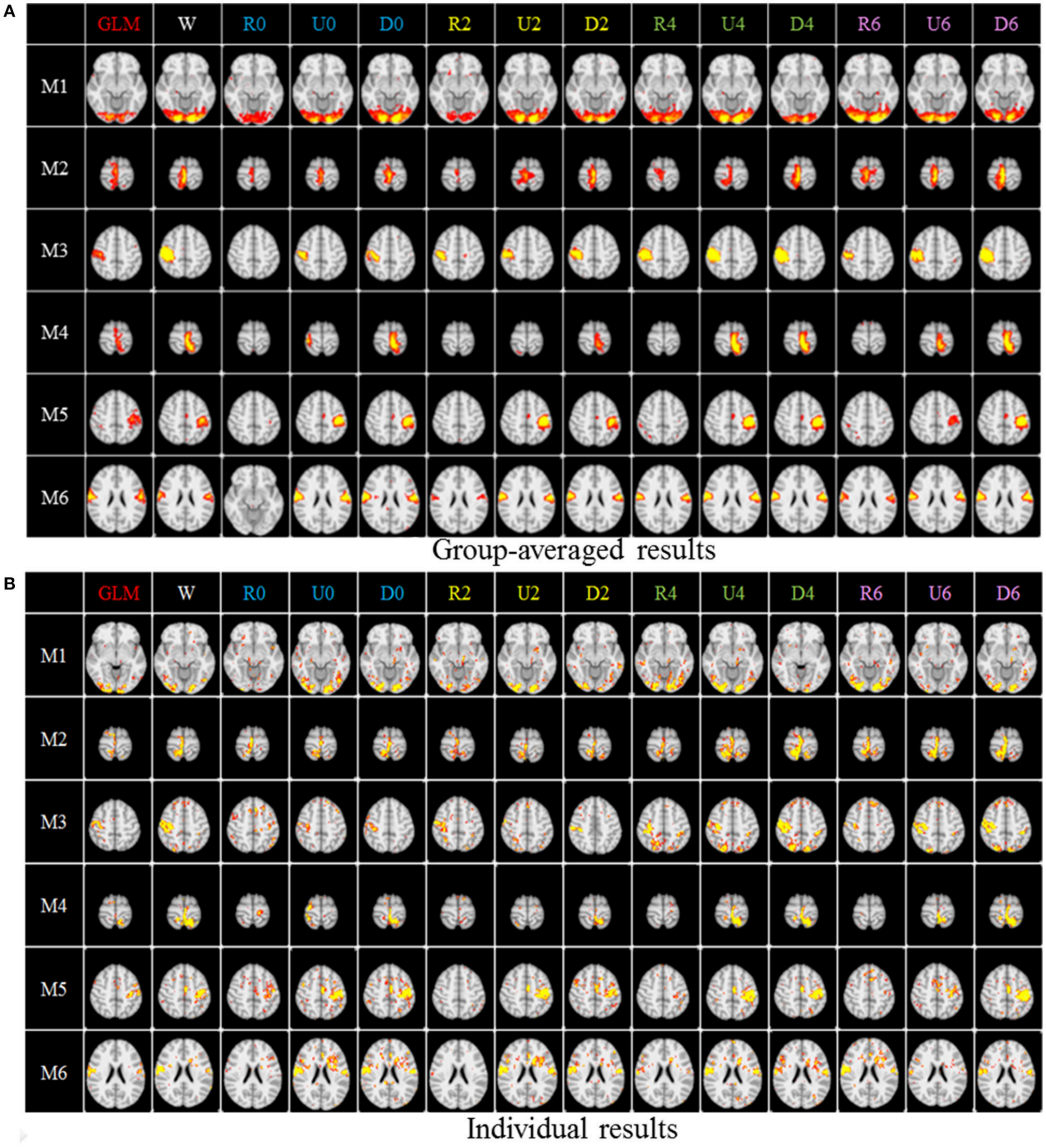
The comparison of spatial task-evoked networks derived from GLM method, no sampling, DICCCOL sampling, random and uniform sampling. “W” denotes the whole-brain signals with no sampling, “Dn” represents n-ring DICCCOL sampling, “Rn” and “Un” represents randomly and uniform sampling, respectively, with the same number of points as the n-ring DICCCOL sampling. “M1” to “M6” represents group-wised saptial map from the six motor tasks, they are visual cue network, left finger network, left toe network, right finger network, right toe network, and tongue Network, repectively. The color scale of spatial maps from GLM ranges from 1 to 6 of Z-score value, and the other color scales range from 0.2 to 5. **(A)** Averaged spatial maps from the group. **(B)** Individual results from one random selected subjects.

After identifying the most correlated dictionary atom with each task stimulus in the above section, we obtained its corresponding row in A, which represents the spatial volumetric distribution of each task-evoked network. Then we compared the spatial patterns of the identified task-evoked networks by the four sampling methods with those derived by GLM. Figure 4 shows the comparison of the derived spatial maps by four sampling methods and GLM method. We can see that the random sampling has the poorest performance, e.g., it is difficult for the R0 sampling (random sampling with 358 sampling points as the 0-ring DICCCOL sampling) to identify the motor task 3, 4, 5, and 6 (M3, M4, M5, M6). In addition, R2, R4, R6 sampling also cannot obtain the correct spatial maps for M4 and M5. The uniform sampling can identify the appropriate networks when it came to R4 with 14,600 sampling points, but U0 and U2 sampling with the fewer sampling points cannot obtain the correct spatial maps for M4. In contrast, the DICCCOL-based sampling can identify all the motor task networks even if the D0 sampling has only 358 sampling points, and it almost has the same performance as no sampling by our visual check. Figure 4 provided the one slice for an overall visual comparison. Quantitatively, we calculated the spatial matching ratio between the spatial volumetric distributions by the four sampling methods and that by the GLM method, as shown in Table 2B. It shows the similar results as Figure 4, that is, DICCCOL-based sampling has the highest spatial matching ratio and random sampling has the lowest SMR values. DICCCOL-based sampling provided more superiority at the level 0 and 2 of sampling, and they (D0 and D2) have higher SMR values (0.41, 0.46) than uniform (0.38, 0.39) sampling and random (0.11, 0.22) sampling, which also can be seen from Figure 4. When the number of sampling points increased to 26,871, the same level of uniform sampling (U6) and DICCCOL-based sampling (D6) had no much difference (0.50 vs. 0.52). We can find that those samplings with the SMR values close to zero in Table 2 have no correct spatial maps in Figure 4 either, e.g., the uniform sampling U0 identifying the M4 (M4, U0) and (M4, U2). We can also see that the DICCCOL-based sampling has the most consistent and balanced performance for M1 to M6. For instance, although the uniform sampling U0 and the DICCCOL-based sampling D0 have the close mean SMR values (0.38 vs. 0.41), if we check into every SMR value for M1 to M6, the DICCCOL-based sampling D0 has the balanced ability to identify all of the motor task-evoked networks but the uniform sampling U0 and random sampling cannot. Meanwhile, we compared their statistical significant differences for detecting each motor task network (M1 to M5), as shown in Table 2B. We can find that, for the M1, M4 and M5 spatial maps, the 0-ring DICCCOL sampling can achieve the same performance with no sampling; for the M2 and M6 spatial maps, the 2-ring DICCCOL sampling can do that, and for the M3, the 4-ring DICCCOL sampling has no significant difference with no sampling. Regarding the uniform sampling, the minimal sampling level is the U2 for detecting the M1, M5 and M6 spatial maps if we require no significant difference with no sampling. And for M4, it must be U6 to meet this requirement. So generally speaking, we need at least the 4-ring DICCCOL sampling to detect all motor task related spatial maps, and if we use the uniform sampling, the level for this is the U6 which has the same sampling points with the 6-ring DICCCOL sampling. Due to the limited space, the statistical comparisons for the random sampling are not shown here. These results further demonstrated the DICCCOL-based sampling can locate the key brain locations from another perspective. At last, we put the averaged SMR values into Table 1A, which is an overall comparison between the four sampling methods.

Table 2**(A)** The Spatial Matching Ratio between each task-evoked network by different sampling methods and that by GLM.

**Table:** 

	**W**	**R0**	**U0**	**D0**	**R2**	**U2**	**D2**	**R4**	**U4**	**D4**	**R6**	**U6**	**D6**
**(A)**													
M1	0.58	0.55	0.59	0.67	0.47	0.65	0.62	0.67	0.63	0.52	0.67	0.64	0.58
M2	0.20	0.08	0.14	0.16	0.06	0.13	0.20	0.09	0.19	0.24	0.17	0.15	0.24
M3	0.83	0	0.56	0.21	0.62	0.61	0.72	0.73	0.81	0.84	0.61	0.84	0.83
M4	0.40	0.00	0.00	0.40	0	0.00	0.30	0	0.33	0.41	0.00	0.44	0.46
M5	0.40	0.02	0.45	0.49	0.00	0.50	0.46	0.04	0.33	0.50	0.09	0.51	0.50
M6	0.34	0.02	0.57	0.55	0.19	0.44	0.45	0.32	0.43	0.44	0.33	0.46	0.48
Mean	0.46	0.11	0.38	0.41	0.22	0.39	0.46	0.31	0.45	0.49	0.31	0.50	0.52

**Table:** 

	**W vs. D0**	**W vs. D2**	**W vs. D4**	**W vs. D6**	**W vs. U0**	**W vs. U2**	**W vs. U4**	**W vs. U6**
**(B)**								
M1	5.63	21.74	9.73	11.80	0.45	7.41	63.64	5.31
M2	3.95	9.25	47.78	19.36	1.35	0.098	41.2	6.64
M3	1.83	0.42	6.4	32.73	0.001	0.023	13.40	67.00
M4	8.37	58.04	13.49	21.81	0.000	0.000	1.01	12.43
M5	10.64	73.84	23.20	11.50	3.84	6.90	9.73	11.80
M6	0.04	21.74	32.73	6.80	2.90	9.74	13.73	9.50

*“W”, “Dn”, “Rn”,“Un,” and “Mn” have the same meanings as those in Figure 4. **(B)** The p-values(× 10^−2^) t-test on SMR. T-test comparisons between no sampling and the DICCCOL/uniform sampling are shown here. The color values denote the *p*-values < 0.05*.

### Other common concurrent networks

**Figure 5:**
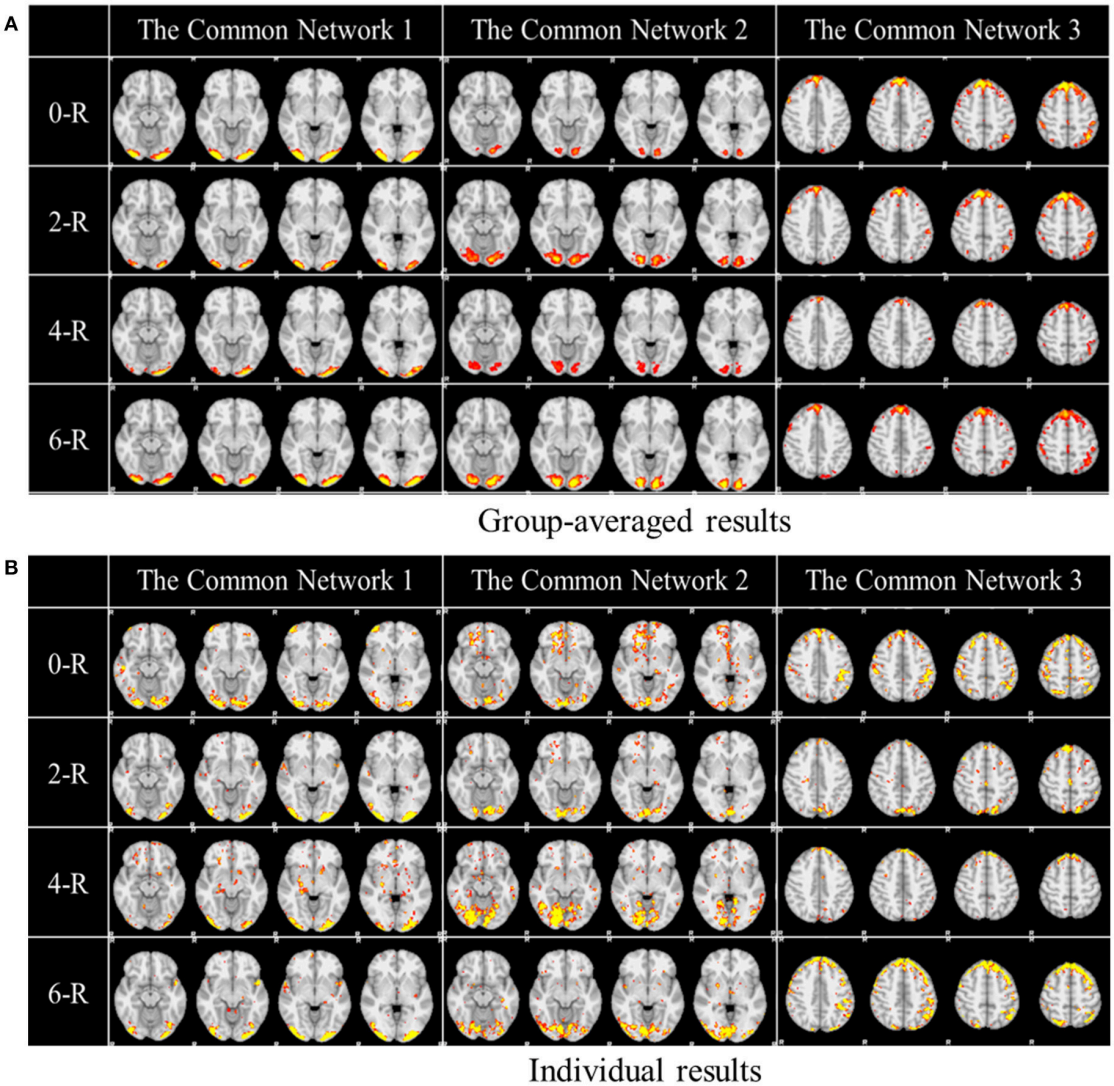
Other common networks identified from the group-wise dictionary learning and sparse representation method and the DICCCOL-based sampling. Here the 3 common networks were identified from 20 subjects, and these 3 common networks are different from the previous 6 task-evoked networks. “0-R” to “6-R” represents 0 to 6-ring DICCCOL-based sampling. The color scale of spatial maps ranges from 0.3 to 3. **(A)** Averaged spatial maps from the group. **(B)** Individual results from one randomly selected subject.

One of the advantages of applying the group-wise dictionary learning and sparse representation to reconstruct brain networks from task fMRI data lies in that it can simultaneously identify the common concurrent/interacting resting state functional networks, which cannot be obtained by the GLM method (Krekelberg et al., 2006; Logothetis, 2008). In order to reveal and interpret these common networks across all the subjects, first, we computed the mean values of all corresponding spatial maps among all the subjects as thresholds in the next step. Here, each spatial map is each row of coefficient matrix A, which is in correspondence among all the subjects given that the dictionary D is common for all. Then we computed the spatial matching ratio between the corresponding spatial maps of any two subjects according to the thresholds above, and then we obtained the averaged SMR value for each corresponding spatial map as the measure of consistency. Finally, we selected the three most consistent spatial maps, as shown in Figure 5. The three common networks from 0- to 6-ring DICCCOL-based sampling are all displayed here, where each common functional network has four representative slices shown. These common networks are also concurrent with those identified networks in section Comparison of Spatial Task-Evoked Networks With GLM Derived Activations. We can see that the common network 1 and the common network 2 are located in two different visual regions as the visual cue network (M1) in the section Comparison of Spatial Task-Evoked Networks With GLM Derived Activations. These two networks can be found in 10 well-defined resting state networks (RSN) templates provided in the literature (Smith et al., 2009).

### Replicability

**Figure 6:**
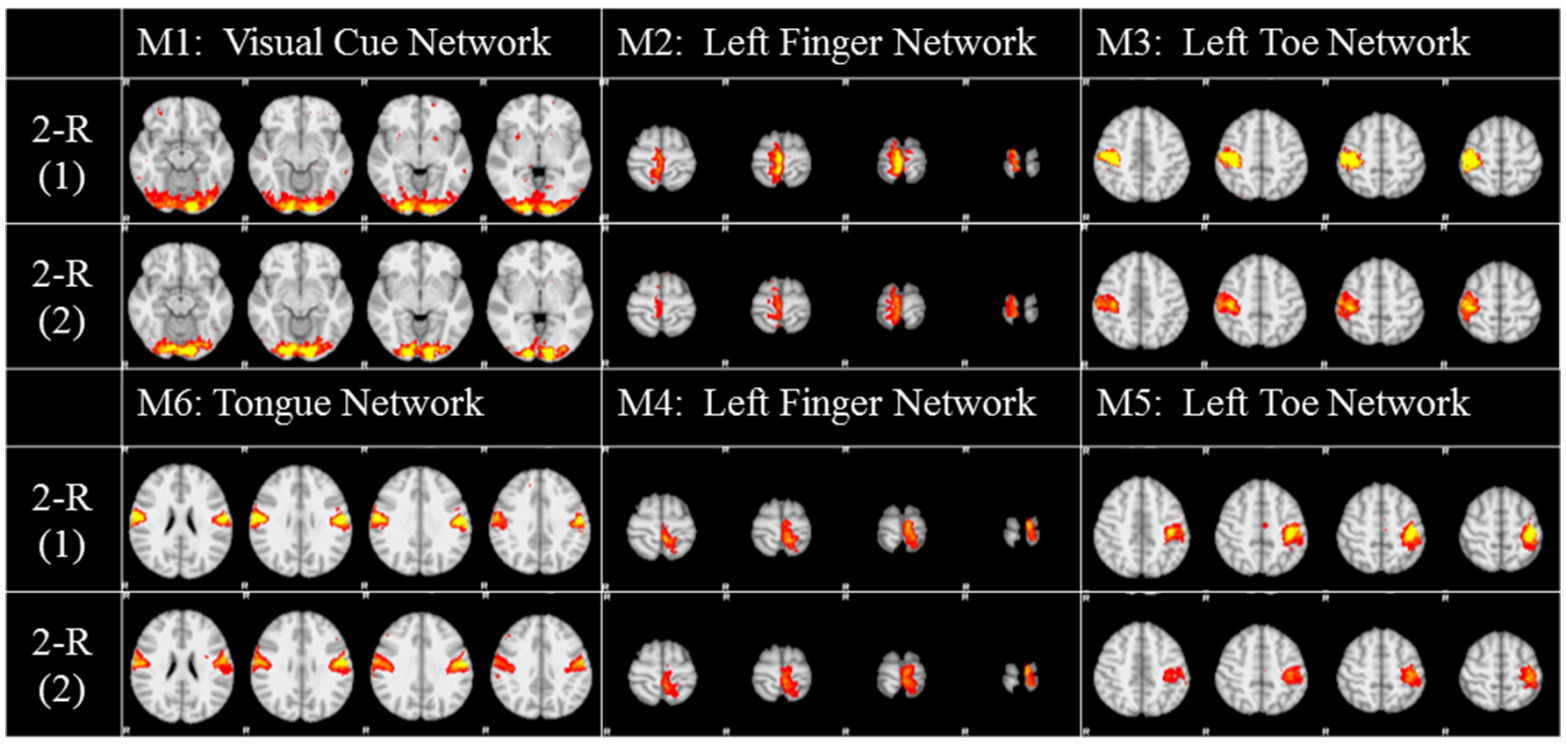
The functional networks identified from the two different groups. Here only the functional networks from the 2-ring DICCCOL-based sampling were shown due to the limite space. “2-R(1)” represents the first run on the previous dataset, “2-R(2)” represents the second run on the new dataset. The color scale of spatial maps ranges from 0.2 to 5.

To investigate the replicability (Patil et al., in review) of the DICCCOL-based sampling and the group-wise dictionary learning and sparse representation method, we applied the same procedure to another separate group of 20 subjects in the HCP task fMRI dataset (Barch et al., 2013). The results are shown in Figure 6. Here, we showed the functional networks via the 2-ring DICCCOL-based sampling. We can see that all the motor task networks can be obtained in the second run, 2-R(2). Moreover, they have very similar spatial maps with the previous networks identified in section Comparison of Spatial Task-Evoked Networks With GLM Derived Activations, which are also presented in the row 2-R(2) for the purpose of comparison. Quantitatively, we computed the SMR between networks of the second run and GLM derived templates, as shown in Table 3A. The second row represents the SMR values of the first run which can be also found in Table 2A, the third row represents the SMR of the second run with GLM templates, *the last* one gives p-values (10^−2^) of t-test comparison between two runs. We can see that we cannot have its performance in the level of the 2-ring DICCCOL sampling although it can detect all the spatial maps. So we compared the statistical difference between two runs via the 4-ring DICCCOL sampling, the SMR values and p-values are shown in Table 3B. We can see that the two runs have no significant difference via the 4-ring DICCCOL sampling, and these results also demonstrated that the proposed method in this paper is replicable. So, with the previous comparison of the PCC and SMR values in section Comparison of Temporal Dictionary Atoms With Task Stimulus Curves and Comparison of Spatial Task-Evoked Networks With GLM Derived Activations, we can conclude that the 4-ring DICCCOL sampling is a safe choice for identifying the task-evoked networks and representing fMRI signals.

**Table 3:** **(A)** The Spatial Matching ratio between the task-evoked networks derived from different datasets via the 2-ring DICCCOL-based sampling.

	**M1**	**M2**	**M3**	**M4**	**M5**	**M6**	**Mean**
**(A) THE SMR VALUES AND** ***p*****-VALUES FROM THE 2-RING dicccol-BASED SAMPLING**							
1st run	0.62	0.20	0.72	0.30	0.46	0.45	0.46
2nd run	0.66	0.60	0.43	0.35	0.57	0.52	0.52
1st run vs. 2nd run	45.84	0.27	0.04	12.30	5.46	8.45	N/A
**(B) THE SMR VALUES AND** ***p*****-VALUES FROM THE 4-RING DICCCOL-BASED SAMPLING**							
1st run	0.52	0.24	0.84	0.41	0.50	0.44	0.49
2nd run	0.61	0.32	0.69	0.36	0.54	0.52	0.51
1st run vs. 2nd run	69.24	9.22	5.85	8.64	17.43	10.83	N/A

*The second row represents the SMR values of the first run which can also be found in Table 2, the third row represents the SMR of the second run with GLM derived templates, and the last one gives the p-values (× 10^−2^) of t-test on SMR values between two runs. **(B)** The Spatial Matching ratio between the task-evoked networks derived from different datasets via the 4-ring DICCCOL-based sampling. Similarly, the last row shows the corresponding p-values (× 10^−2^) of t-test on SMR values between two runs. The color values denote the p-values < 0.05*.

### Comparison of time costs

**Figure 7:**
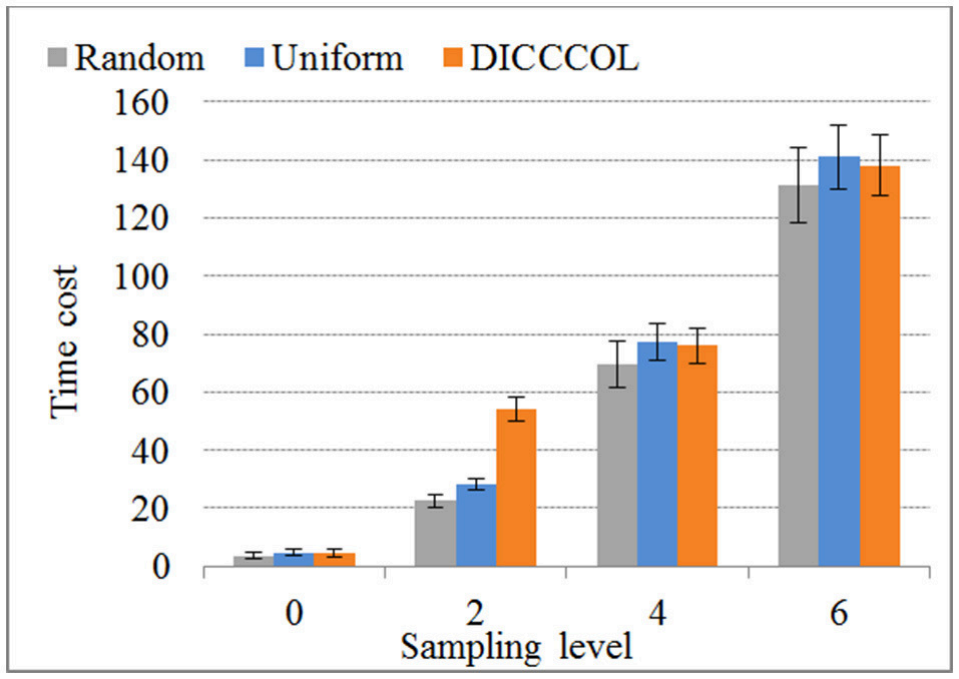
The time cost of differnt sampling methods. “0–6“ represents the sampling level which has the same number of sampling points with 0 to 6 ring DICCCOL-based sampling.

We compared the time costs for the four sampling methods. Since the dictionary learning step is a major part and it costs more time than the sparse coding step (which is fixed) (Mairal et al., 2010), the difference of time cost heavily depends on the number of task fMRI signals given that the dictionary size is fixed (here the size is 400). We therefore only compared the time cost of dictionary learning step, as shown in Figure 7. We can see that the time cost increased with the increase of the number of sampling points, and it was almost not affected by sampling methods. As an example, a 4-ring DICCCOL-based sampling with robust performance has the averaged 14,600 signals (each subject contains around 2.4 × 10^5^ whole-brain fMRI signals). The time costs of no sampling, DICCCOL-based sampling, uniform sampling, and random sampling methods for 20 subjects are 1,953, 69.7, 77.4, and 76.1s, respectively. It is obvious that DICCCOL-based sampling with the 6% sampled points is approximately 28 times faster than no sampling, without sacrificing much accuracy for sparsely representing the whole brain's task fMRI signals. If we have stricter need for time than accuracy, we can adopt the 0 or 2-ring DICCCOL-based sampling. Otherwise, we can use the 4/6-ring DICCCOL-based sampling. Notably, here we aggregated the 20 brains' fMRI data into one data matrix to test and validate. In fact, one can aggregate more brains' fMRI data into the input data matrix, which depends on the memory space availability. Then less time cost and higher accuracy can be expected, which is very useful in an fMRI big data context. In conclusion, the proposed DICCCOL-based sampling, combined with the group-wise dictionary learning across individual subjects, achieved 15–400 times speed-up, allowing for appropriate accuracy to identify the functional brain networks.

### Setting of parameters in dictionary learning and sparse coding

**Figure 8:**
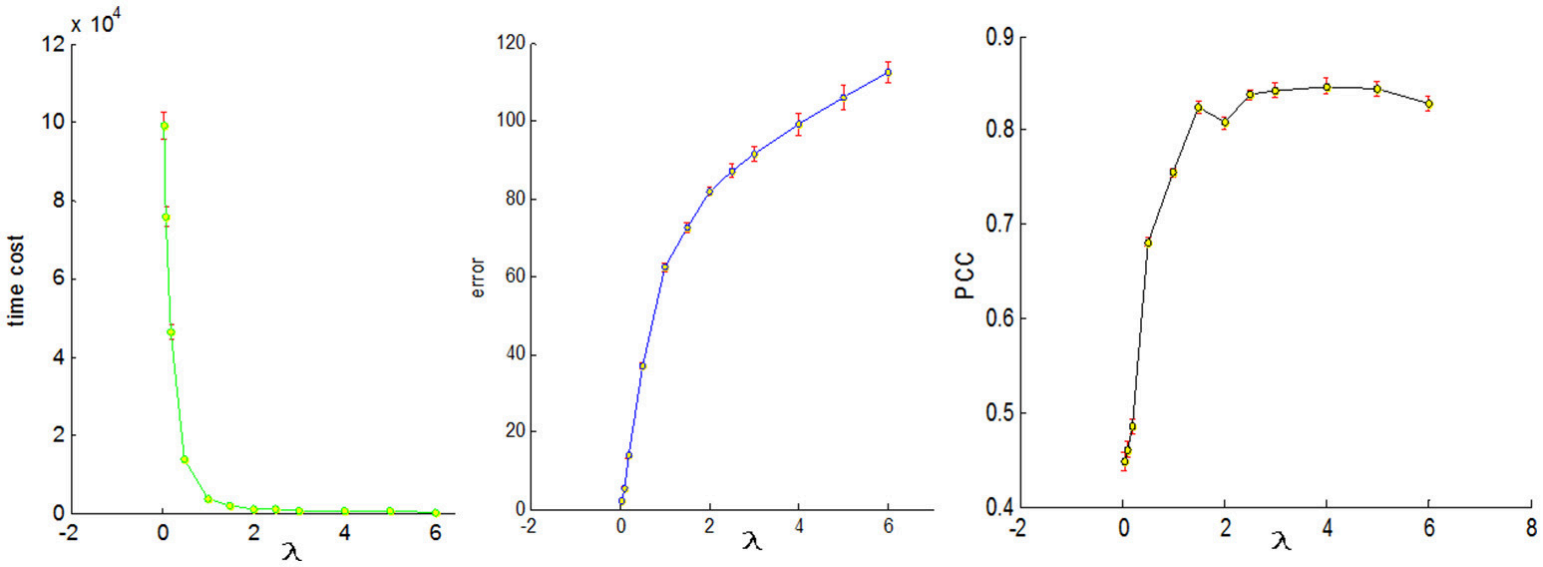
The three change curves with varying λ. These curves are the averaged representation errors, the computing time costs, and the averaged Pearson's correlations between the 6 task stimuli and the corresponding most matched dictionary atoms, respectively.

To conduct a fair comparison, we chose the same parameters for all of the four sampling methods, that is, the number of dictionary atom is 400, the sparsity regularization parameter λ = 1.5, and we set the number of iterations for whole brain's signals as 4. The justification for the selection of the number of dictionary atoms can be referred to Lv et al. (2014b). Basically, we have a wide range of choices from 300 to 500, and the number within this range can reliably uncover the dominant basis components that can well represent the fMRI signals. The online dictionary learning and sparse coding algorithm learns the dictionary batch by batch, but not by whole brain data. We set the number of iterations for each batch as 100, which plays an important role in determining the representation error. We set the batch size as the number of signals times 4 divided by the number of iterations so that the whole signal matrix was employed 4 times, thus providing a foundation for fair comparison. In order to determine the sparsity regularization parameter λ, we plotted the change of time cost, representation error and Pearson's correlation curves with the λ, as shown in Figure 8. The time cost (in seconds) was the time to compute the common dictionary of the 20 subjects using one computing core, and the representation error (in dB) was the averaged values among 20 subjects. The Pearson Correlation Coefficients (PCC) between the six task stimuli and their corresponding most matched dictionary atoms were also the averaged values of the six PCCs among 20 subjects, with the range of PCC from 0 to 1. We found that the averaged PCCs increased as the λ rose to 1.5, and then tended to be stable nearby 0.8, which means an adequate high Pearson correlation between two signals (this fact can be seen from the Figure 3). Meanwhile, the representation error and time cost always increased and decreased with the rise of the λ, respectively. Therefore, the λ value was a trade-off result in consideration of the three performances of dictionary learning and sparse representation. We adopted the same set of parameters for all four sampling methods.

## Discussion

In this work, we presented a novel structurally guided fMRI signal sampling scheme for effective group-wise dictionary learning and sparse representation of whole brain task fMRI signals. The comparative experiments demonstrated that DICCCOL-based sampling combined with group-wise dictionary learning achieved 15–400 times speed-up for signal representation, and we think that the 4-ring DICCCOL sampling is a safe choice without significant loss for identifying the task-evoked networks and representing fMRI signals. Furthermore, the group-wise strategy of dictionary learning and sparse representation can efficiently and easily identify other concurrent resting state functional networks from task fMRI data which cannot be detected by the traditional GLM method. The DICCCOL-based sampling method is different from the previous reduction techniques which use the mathematical theory, as it leveraged the structural information of brain and was demonstrated more effective. This proposed method is significant for dealing with large scale fMRI data, especially when functional networks analysis becomes an important step for discovering the underlying organization structures and meaningful dynamic patterns from the vast amount of fMRI snals (Li et al., 2016). We could apply this method to a distributed and parallel computing environme*nt* (Kiar et al., 2017), as the DICCCOL-based sampling of each brain can be in parallel. The dictionary learning algorithm is also parallelized in its code, and the pre-processing of DTI including DICCCOL and fMRI can be parallelized, so the processing of DTI and DICCCOL is not a time-cost problem in a distributed and parallel computing environment. In the future, we plan to further evaluate and validate this method using other task fMRI datasets, and compare the DICCCOL-based sampling method with other more advanced signal sampling methods.

## Ethics statement

This study was carried out in accordance with the recommendations of Name of Duidelines, Name of Committee with written informed consent from all subjects. All subjects gave written informed consent in accordance with the Declaration of Helsinki. The protocol was approved by the Name of Committee.

## Informed consent

This study used a public fMRI dataset that was acquired by the Human Connectome Project (HCP). The informed consent was obtained during the HCP project.

## Author contributions

BG contributed to designing and analyzing the experiments, summarizing and visualizing the results, and writing the manuscript. XL and XJ contributed to preparing the data, and the revision of the manuscript. YS contributed to analyzing the experiments and the revision of the manuscript. TL contributed to directing the whole project.

### Conflict of interest statement

The authors declare that the research was conducted in the absence of any commercial or financial relationships that could be construed as a potential conflict of interest.
